# Exploring meaning of participation in a clinical trial in a developing country setting: implications for recruitment

**DOI:** 10.1186/1745-6215-12-S1-A114

**Published:** 2011-12-13

**Authors:** Joanna Reynolds, Peter Mangesho, Lasse S Vestergaard, Clare Chandler

**Affiliations:** 1 London School of Hygiene & Tropical Medicine, UK; 2 National Institute for Medical Research, Amani Centre, Tanzania; 3 University of Copenhagen, Denmark

## Objectives

This study aimed to explore the experiences of people participating in a clinical trial in Tanzania. We sought to understand the meaning attached to participation and how experiences of being in the trial related to participants’ original motivations for consenting, in order to explore appropriate strategies for recruitment in a developing country setting.

## Methods

We designed a qualitative study alongside a clinical observational trial of the efficacy and safety of artemisinin-based combination therapy (ACT) for malaria in patients concomitantly receiving antiretroviral therapy (ART) for HIV in Muheza, Tanzania. Focus-group discussions have been held with HIV-positive and HIV-negative people who have participated in the trial, and with HIV-positive people who were screened but who did not participate. This data has been triangulated with in-depth interviews with staff conducting the trial and delivering HIV care at the hospital where the trial was conducted. Data is being analysed using an iterative, line-by-line approach based on the principles of grounded theory, to identify units of meaning and develop themes and constructs from the data.

## Results

Analysis to date of eight FGDs and IDIs indicates a disconnect between the information given to trial participants in the recruitment and consent process and their understanding of the trial and its aims, with a few participants stating they did not realise they were part of a research study. This reflects, and may be attributable to trial participants’ frequent conflation of the clinical encounter - testing for malaria - with the research encounter - the recruitment process - and an inability to distinguish between these as separate events. When describing recruitment, many participants framed their narrative around clinical events such as the malaria test and seeking treatment, the latter being considered the most important reason for joining the study. The clinical context in which participants were screened and recruited appeared to influence their ability to interpret information about the trial and expectations for what may happen to them, raising questions about the nature of ‘informed consent’ in the recruitment process.

Participants reported overwhelmingly positive experiences of participating in the trial, largely based around their access to numerous tests and free treatment, as well as reimbursement for transport and telephone costs. Being part of the trial was frequently conceptualised as receiving a ‘service’, valued chiefly for enabling participants to be observed and to know their health status. The perceived value of this ‘service’ was reflected in many participants’ reports of encouraging friends and family to attend for malaria testing, in order to access the service associated with the trial. Although this indicates again some confusion between clinical and research activities, it appeared that the ‘enactment’ of the trial – giving and receiving the service – offered the social space in which participants were likely to raise any concerns about aspects of the trial. Such concerns included questioning the ‘true’ aim of the study, and fears over blood taking. This suggests that it is within a relationship of interaction with trial staff and activities that comprehension about the meaning and value of participation can begin to emerge, thus highlighting the potential shortcomings of the standard recruitment process.

## Conclusions

The findings from this study raise questions about how information is presented and used in the recruitment and consenting process for a trial, particularly one located within an existing clinical context. Recruitment strategies should take into consideration when, where and how information will be conveyed to participants, and explore likely expectations of these contexts which may shape how and if information is interpreted and utilised by potential participants. The findings also indicate that the meaning participants place on participation, its risks and benefits, develops largely through enactment of the relationship with trial staff and activities, rather than through the consent process. To ensure better informed consent, researchers could seek to activate this relationship earlier on in the trial process, for example through meaningful community engagement activities prior to recruitment.

This study has also underscored the value participants in this low-resource setting place on engaging with a ‘service’ of health care provision, wherein they perceive their health status and needs to be the focus of trial activities. This service is an influential motivator for continuing to engage with a trial, beyond more material compensations such as money for transport. These findings also suggest the potential for word-of-mouth strategies to aid informed recruitment in contexts where the perceived benefits of participation are highly valued amongst the local community.

Recruitment and continued participation in trials involve logistical and ethical challenges. Understanding values and concerns of participants can pose practical solutions.

